# Incorporating Community Case Management in Risk-Based Surveillance for Malaria Elimination in the Dominican Republic

**DOI:** 10.4269/ajtmh.24-0404

**Published:** 2025-01-21

**Authors:** Isabel Byrne, Luca Nelli, Keyla Ureña, Luccène Désir, Claudia Hilario Rodriguez, Nicole Michelén Ströfer, Justin T. Lana, Gregory S. Noland, Manuel de Jesús Tejada Beato, Jose Luis Cruz Raposo, Chris Drakeley, Karen E. S. Hamre, Gillian Stresman

**Affiliations:** ^1^ Department of Infection Biology, London School of Hygiene and Tropical Medicine, London, United Kingdom;; ^2^ School of Biodiversity, One Health & Veterinary Medicine, University of Glasgow, Glasgow, United Kingdom;; ^3^ Centro de Prevencion y Control de Enfermedades Transmitidas por Vectores y Zoonosis, Ministerio de Salud Pública, Santo Domingo, Dominican Republic;; ^4^ The Carter Center, Atlanta, Georgia;; ^5^ Malaria Team, Clinton Health Access Initiative, Boston, Massachusetts;; ^6^ Department of Epidemiology, College of Public Health, University of South Florida, Tampa, Florida

## Abstract

As countries strive for malaria elimination, it is crucial to gather sufficient evidence to confirm the absence of transmission. Routine surveillance data often lack the sensitivity to detect community transmission at low levels. In the Dominican Republic, community health workers (CHWs) have been deployed in malaria foci to perform active case detection. This study aimed to assess the added value of CHWs in enhancing the health system’s malaria detection capabilities. Freedom from infection (FFI) is a statistical framework designed to demonstrate the absence of malaria by using routinely collected health data. We adapted this framework to include CHW data, estimating their contribution to the health system’s malaria detection ability. The model was applied to facility and CHW data from 33 facilities across nine provinces in the Dominican Republic, covering the period from January 2018 to April 2022. The likelihood that a facility’s catchment population is free from malaria infection (*P*free) was achieved in 52% of facilities by using only routine data, sustained for an average of 13 months. With the addition of CHW data, 88% of facilities reached *P*free, sustained for an average of 37 months. Incorporating CHW data enhanced the precision of model estimates by over 500-fold. The study demonstrated the near absence of malaria in several facility catchment populations. It highlighted the importance of community case management in supplementing routine surveillance, thereby improving the precision of malaria transmission estimates. These findings support the further application of the FFI framework to accelerate progress toward malaria elimination in the Dominican Republic.

## INTRODUCTION

The island of Hispaniola, comprised of the Dominican Republic and Haiti, remains the final island in the Caribbean where malaria is endemic.^1^ The Dominican Republic is named by the WHO as one of the countries to eliminate malaria by 2025.^2^ In line with this goal, the Ministry of Health is developing a comprehensive nationwide malaria elimination plan. This plan includes measures to strengthen the surveillance system, including community case management (CCM) consistent with the WHO certification criteria.^3^^,^^4^ The nation has reported fewer than 1,500 indigenous cases annually since 2011, with *Plasmodium falciparum* infections making up >99%.^4^ Cases are declining: 826 indigenous cases were reported in 2020,^3^ while 320 were reported in 2022.^5^ The burden of malaria is significantly lower in the Dominican Republic than in neighboring Haiti, with 14,757 (98%) of all 15,094 confirmed malaria cases on the island of Hispaniola in 2022 reported from Haiti.^5^ Historically, malaria transmission in the Dominican Republic has been low and generally focused in rural areas, including the Haitian border, and in regions populated by migrant agricultural laborers, suggesting importation from Haiti.^6^^,^^7^ Recently, however, transmission has been interrupted in the cross-border focus in Ouanaminthe-Dajabon.^3^ In 2014, repeated outbreaks began in the urban and semiurban areas of Santo Domingo Province and the National District (Distrito Nacional). The causes for these outbreaks remain unknown but may be related to migration into the city from areas of endemicity,^8^ with the vast majority of cases detected in Dominican nationals.

Subsequent to the onset of these outbreaks, the National Malaria Control Program (NMCP) was decentralized in 2016, causing a shift in primary malaria programming to local health districts and the creation of new roles, including community health workers (CHWs).^8^^,^^9^

The CHWs were introduced to perform active surveillance within the communities most impacted by the outbreaks. Community health workers perform active case detection (ACD) through door-to-door screening in their designated area of influence, 5 hours a day, 5 days a week. Community health workers are expected to visit at least 40 houses per day worked (200 houses per week). Individuals suspected of fever are tested by rapid diagnostic test (RDT), followed by blood films for confirmatory diagnosis by microscopy, and offered treatment based on their RDT result.^8^ Since 2019 in the Dominican Republic, CHWs organized in networks and are associated with a health facility but separately report the individuals they attend to and suspect, test, and treat for malaria. Passive case detection (PCD) in the Dominican Republic is performed primarily at health facilities and on some occasions when a CHW is visited in their home by a community member. Microscopy is used as the gold standard for malaria testing at health facilities,^8^ with some facilities also offering RDTs.^4^

As cases decrease and the country strives toward its malaria elimination goals, sufficient evidence is required to confirm the elimination of malaria. A country may request WHO certification as malaria-free only after reporting zero indigenous cases for 3 consecutive years and with sufficient surveillance data to support the absence of transmission.^10^ To achieve this, an effective surveillance system is essential. Measuring the absence of a disease or infection is a challenge, as it involves proving a negative^11^; routine statistical methods are impractical in the absence of a perfect diagnostic applied to an entire population.^12^^,^^13^

“Freedom from infection” (FFI) is a suite of risk surveillance statistical methods initially developed in the context of veterinary epidemiology. The FFI approach provides established methods for measuring the probability of having achieved elimination. Its application to malaria and other human disease systems has recently been described.^11^^,^^14^^,^^15^ Briefly, the malaria FFI model consists of a statistical framework designed to 1) quantify the likelihood that a surveillance system can detect infections surpassing a specified threshold, a capability referred as surveillance system sensitivity (SSe), and 2) assess the likelihood that the absence of reported cases genuinely indicates no malaria transmission, referred as the probability of freedom from malaria (*P*free).^11^^,^^15^ Moreover, the methodology utilizes statistical inference to identify key parameters within the surveillance system which are most influential to SSe and *P*free estimation.

Community health workers are key components of many malaria control programs and are used to implement CCM to ensure prompt access to malaria testing and treatment.^16^ Community health worker activities constitute a potentially important source of information that could supplement the routine facility-based surveillance data; indeed, determining the value added to malaria surveillance would be an important element to support justifying such CHW programs in elimination settings.

Here, we apply the full FFI model framework to PCD in an elimination setting for the first time. Additionally, we have adapted the FFI model to incorporate the data from CCM. We aimed to estimate the added value of CCM to the health system’s ability to detect malaria by quantifying the change in model estimate precision when CHWs are active within a health district. We also sought to determine which key parameters within the surveillance system are most influential to SSe and *P*free estimation, which may inform decision-making on specific spatial regions or branches of the surveillance system that could be targeted for improvement.

## MATERIALS AND METHODS

### Sampling strategy.

**Figure 1.**
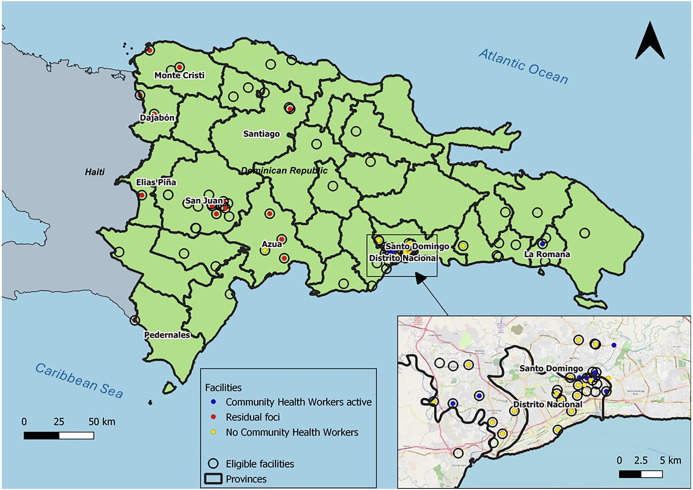
Map of Dominican Republic and border with Haiti, including an inset map of the Santo Domingo area. Selected facilities are represented by colored circles. Red circles represent catchments in residual foci of transmission, blue circles represent facilities with active CHWs, and yellow circles represent randomly selected eligible facilities (eligibility defined as provision of rapid diagnostic test and/or microscopy malaria testing). Unselected facilities which were eligible are represented by black circles. Provinces from which data were collected are labeled. Santo Domingo and Distrito Nacional inset map details were provided by OpenStreetMap (basemap data copyrighted by OpenStreetMap contributors and available from https://www.openstreetmap.org).

The selection of facilities was conducted with the NMCP, and facilities which had malaria testing capacity through microscopy and/or RDT were eligible for selection. First, to assess the SSe in facilities within residual foci of transmission, health facilities in border provinces and in provinces which are expected to have high Haitian migrant populations^6^^,^^7^ were selected. These included Monte Cristi, Dajabón, Santiago, Elias Piña, San Juan, Azua, Pedernales, and San Pedro de Macoris. All 14 eligible health facilities in these provinces were selected. Second, to quantify the impact of CHWs on the estimated SSe and FFI, provinces where CCM was being implemented were selected. At the time of sample selection, CHWs operated only in Santo Domingo and Distrito Nacional. Of the 55 eligible facilities in this area, 34 were randomly selected and then stratified by whether at least one CHW was operating out of that facility. Ten facilities with CHWs operating and 24 facilities with no CHWs were sampled. In total, 48 facilities were selected across the country, as illustrated in Figure 1.

### Data collection.

Three sources of data were required for this study: 1) health system interviews targeting clinicians and CHWs; 2) PCD routine malaria surveillance data from the health facility; and 3) ACD data from CHWs. All data were collected on Android phones using the Carter Center OpenDataKit-based NEMO software.^17^

Health system questionnaires were developed with the NMCP and administered to CHWs and health facility staff by data collectors to assess the health system’s capacity to detect malaria cases. The staff member responsible for dealing with malaria testing and case management in each facility was targeted for interviews. Briefly, the questionnaire collected data related to the supply chain of malaria testing and treatment commodities, the size of the population in the facility catchment area, patient management, staff technical capacity and training, case definitions, and standard procedures for diagnostics applied in the facility. A detailed list of questions is reported in Supplemental Table 1.

Data from passive case detection routine malaria surveillance from January 2018 through April 2022 were extracted from the paper-based registries at each facility. Data were condensed to monthly counts. The recorded numbers of people attending the clinic (for any condition), presenting with fever, suspected of malaria, tested for malaria, and confirmed positive for malaria were also collected.

The ACD data collected from CHWs are not stored at health facilities and were provided by the NMCP.

## STATISTICAL ANALYSES

The FFI analytical framework^15^ was used to assess SSe. This involved estimating the probability of an individual seeking medical care ($P_{\mathrm{SEEK}}$), the probability of a clinician suspecting malaria ($P_{\mathrm{SUSPECT}}$), and the probability of being tested for malaria ($P_{\mathrm{TEST}}$). Briefly, a malaria case management cascade is used to model the flow that an individual takes through the system for an infection to be detected in the routine surveillance system. Subsequently, the calculated probabilities and overall cascade were used to estimate the SSe, here defined as the difference between the observed and expected numbers of cases in the community that should be detected. A detailed description of the model is provided in Supplemental Appendix 1. A high degree of overlap between the observed and expected numbers of cases indicates robust system sensitivity in identifying malaria infections, contingent on a predetermined threshold. Subsequently, the calculated SSe informs the estimation of the *P*free, reflecting the likelihood that the catchment population is free from malaria infection.

Previous models were developed using longitudinal PCD data; here, we have assessed the added value of CCM by estimating the additional SSe gained within health system components when CHW data were combined with the routine facility data. The additional steps to the FFI model described by Ahmad et al.^14^ and Nelli et al.^15^ are in Supplemental Appendix 2. Briefly, data collected in health system interviews were analyzed in a regression framework to determine the association with $P_{\mathrm{SEEK}}$, $P_{\mathrm{SUSPECT}}$, and $P_{\mathrm{TEST}}$. Several of the parameters have inherent biological limits (e.g., the probability that someone has fever or the sensitivity and specificity of the diagnostic tests) and therefore are independent of the health system and were parameterized according to the literature.^15^ Accurate catchment population data are crucial for applying the FFI model. However, we encountered a lack of recorded catchment population information for 19 of these facilities. To address this, we used data from the Global Human Settlement Layer (GHSL)^18^ to determine the total population within a 5-km radius surrounding each facility. Subsequently, a simple linear regression model was developed, using the GHSL-derived population figures to predict the catchment populations for the 15 facilities where such data were already available. This regression model was then applied to estimate the missing catchment population figures for the 19 facilities lacking direct data.

To quantify the change in the precision of model estimates when CHW data were included, we calculated the difference in the credible interval range of the confidence bands between the model estimations of malaria cases per 10,000 people from PCD data and PCD plus CHW data. The credible interval range was calculated as the lower limit of the credible interval subtracted from the upper limit of the credible intervals for estimations. It was calculated for each time point at each health facility where CHW data were available. The difference in range is used here as a proxy for the difference in the precision of model estimates when CHW data were added to the model. Where the difference in precision is negative, the estimates of precision have improved by the addition of CHW data and the bands have shrunk. Conversely, where the difference in precision is positive, there has been a loss in precision of the estimates, corresponding to the widening of the bands. Additionally, we calculated the quantity of change, or fold change, in the precision of estimates when CHW data were added. This was calculated as the average range in credible interval for PCD data divided by the average range in credible interval for PCD plus CHW data.

## RESULTS

### Overview of data collected.

Data were collected from health facilities from May through June 2022.

For the health system interviews, 47 out of 48 hospitals visited provided interviews. The majority (64%) of the responses were from doctors, with 12 from laboratory technicians and the remainder from nurses, administrators, and malaria supervisors. The results of the health system interviews for health facilities and CHWs are presented in Table 1. Community health workers consistently reported higher rates of recording of data, testing, provision of antimalarial drugs, training, and supervision. The length of stockouts ranged from 1 to 12 months for RDT and 3 to 8 months for microscopy.

**Table 1:** Summary of results of health system interviews for health facilities and CHWs

Parameter	Health Facility (%)	CHW (%)
Number of facilities visited	47	13
Hospital	24	1
Primary care center	22	12
Diagnostic center	1	0
Number of interviews conducted*	47	20
Record suspected malaria cases	31 (66)	20 (100)
Record individuals tested for malaria	31 (66)	20 (100)
Record confirmed malaria cases	33 (70)	20 (100)
Provide RDT testing^†^	40 (85)	19 (95)
Experienced RDT stockout in past 12 months^†^	21 (53)	1 (5)
Provide microscopy testing^†^	14 (30)	20 (100)
Perform microscopy cross-checking with reference laboratory^†^	12 (86)	NA
Experienced microscopy stockout in past 12 months^†^	2 (14)	20 (100)
Provide antimalarial drugs	19 (40)	20 (100)
Experienced antimalarial drug stockout in past 12 months	1 (.05)	3 (15)
Have copy of national treatment guidelines/SOP for case management	25 (53)	18 (90)
Have staff to conduct testing	44 (93)	NA
Staff had training on malaria diagnosis/case management in past 2 years	27 (57)	20 (100)
Reported supervisor visit in past year	25 (53)	18 (90)
Received training on completing registry forms in past year	9 (19)	16 (80)

CHWs = community health workers; NA = not applicable; RDT = rapid diagnostic test; SOP = standard operating procedure.

* The total numbers of interviews were 47 from health facilities and 20 from CHWs. The CHWs interviewed in this study performed active case detection only.

† Health facility percentages for RDT, microscopy, drug stockout and cross-checking were calculated from the number of health facilities providing them.

For the PCD collection, 33 of the 47 health facilities had malaria registry data available from January 2018 through April 2022. Of the 52 months for which PCD data were collected, only one health facility had data available on the number of suspected, tested, and confirmed cases every month when the study teams were present, and no facilities had data available for attendance every month. The number of months that facilities had data available on individuals suspected, tested, and confirmed for analysis ranged from 1 to 51, and the number of months that facilities had data available on attendees ranged from 1 to 49. The health area directorate in the capital area of Santo Domingo and the Distrito Nacional had facilities where the data were more consistently available when study teams were present.

For the ACD data collection, CHW data were retrieved for 13 facilities for January 2019 through April 2022. Community health workers prior to the reorganization of 2019 were not associated with specific health facilities. Therefore, the CHW data which were collected before this reorganization could not be easily integrated into the facilities-based FFI model. Of the 40 months for which data were collected, no CHW reported data every month. The number of months with no data reported for attendees or suspected, tested, and confirmed cases ranged from 9 to 12, with some periods of missing data consistent over multiple facilities.

### Estimating SSe.

**Figure 2.**
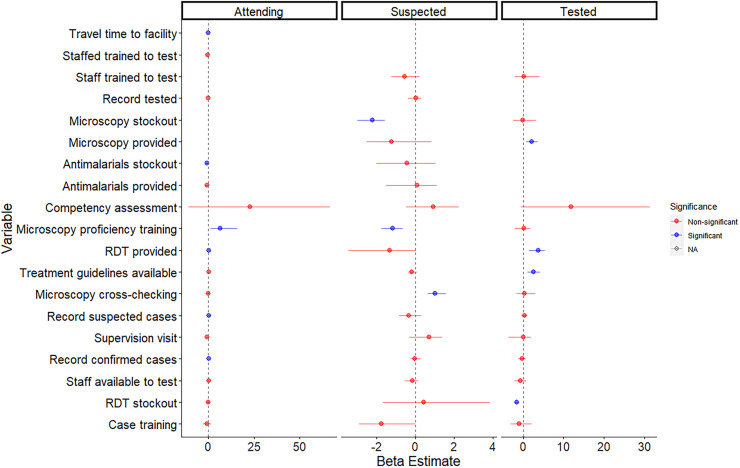
Forest plot of results from Bayesian models for probability of care-seeking (attending), probability of being suspected of having malaria (suspected), and probability of being tested for malaria (tested) as a function of covariates obtained through questionnaires at health facilities. Circles represent beta estimates (mean of posterior distribution), and arms (horizontal lines) represent lower credible intervals and upper credible intervals. Variables denoted in red have posterior distributions which span 0. Variables denoted in blue have posterior distributions which do not span 0. NA = non-applicable; RDT = rapid diagnostic test.

The results of the regression modeling to determine the association between health system interview parameters and $P_{\mathrm{SEEK}}$, $P_{\mathrm{SUSPECT}}$, and $P_{\mathrm{TEST}}$ for malaria are presented in Figure 2 and Supplemental Table 2. The results show that individuals were more likely to seek care at facilities where RDTs are provided (β 0.52, 95% CI: 0.35–0.74), microscopy proficiency training is provided (β 6.51, 95% CI 1.51–15.91), and records of confirmed (β 0.24, 95% CI: 0.05–0.43) and suspected (β 0.48, 95% CI 0.09–0.91) cases are kept. Facilities are less likely to have individuals seek care if they experienced antimalarial stockouts in the past 12 months (β −0.63, 95% CI: −1.23 to −0.11) and if patients experienced longer travel times (β −0.07, 95% CI: −0.14 to −0.03). An individual who entered a facility was more likely to be suspected of malaria if routine monthly cross-checking of microscopy slides with the reference laboratory was performed (β 1.02, 95% CI: −0.64 to 1.58). Factors which increased the probability of being tested at a facility if suspected of malaria included the provision of RDTs (β 3.67, 95% CI: 1.45–5.38) and microscopy testing (β 2.11, 95% CI: 0.84–3.65) and the availability of national malaria treatment guidelines (β 2.59, 95% CI: 1.13–4.19). Facilities were less likely to test if they had experienced RDT stockouts in the last 12 months (β −1.55, 95% CI: −2.52 to −0.98).

### FFI model results.

The *P*free results for each of the facilities ranged from 0 to 1. Seventeen of 33 (52%) health facilities achieved a *P*free equal to 1 with PCD data alone. These facilities maintained *P*free for a range of 0–52 months, with an average of 13 months (SD ±19). When paper data were unavailable to review at the health facility, wide uncertainty in the estimated SSe often resulted, but the results based on the health facility interviews and routine data suggest that in some facility catchments, local malaria elimination is likely to have occurred.

**Figure 3.**
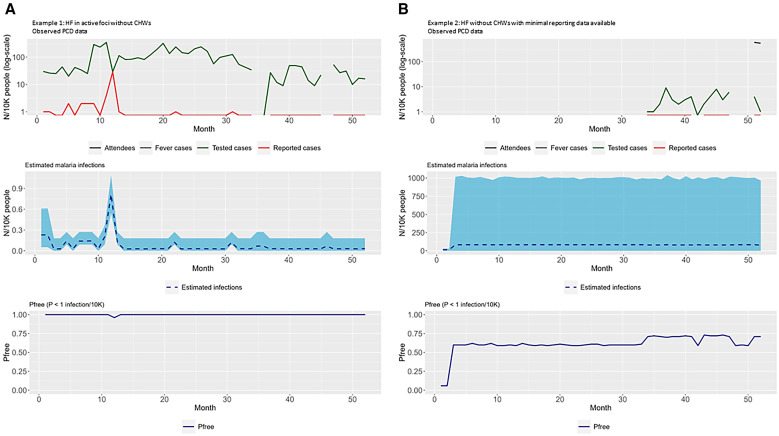
Examples of the freedom from infection (FFI) modeling outputs for routine health facility (HF) data. (**A**) Health facility in active foci without community health workers (CHWs). (**B**) Health facility without CHWs with minimal reporting data available. The top panels show the raw passive case detection (PCD) data: black, attendees; blue, suspected (fever); green, tested; red, confirmed. The middle panels show the estimated malaria cases represented by a blue dashed line and the corresponding 95% CI in light blue. The bottom panels show the estimated probability of freedom from malaria infection (PFree), represented by a dark blue line.

**Figure 4.**
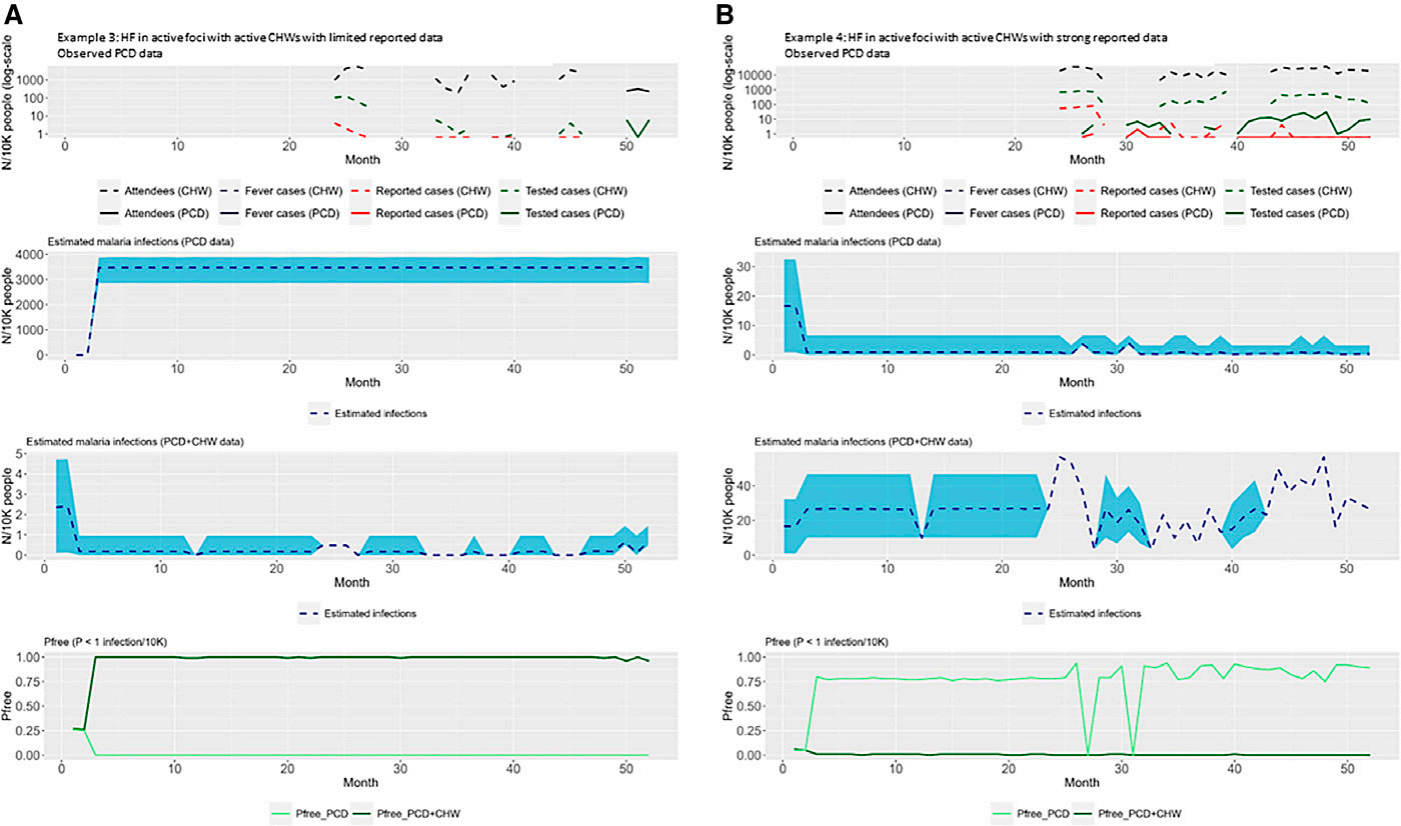
Examples of the freedom from infection (FFI) modeling outputs when community health worker (CHW) data are combined with routine health facility (HF) data. (**A**) Health facility in active foci with active CHWs with limited reported data. (**B**) Health facility in active foci with active CHWs with strong reported data. The top panels show the raw data, with passive case detection (PCD) data represented by a solid line and CHW data represented by dashed lines. Black, attendees; blue, suspected; red, confirmed. The upper middle panels show the estimated malaria cases with the PCD-only model. The lower middle panels show the estimated malaria cases with the PCD plus CHW model. The bottom panels show the estimated probability of freedom from malaria infection (PFree) with the PCD-only model (light green) and the PCD plus CHW model (dark green).

Two health facilities without active CHWs were selected to highlight the importance of strong routine data collection and reporting on the sensitivity of a health system to detect cases should they exist (Figure 3). To highlight the changes in SSe and *P*free when CHWs are active within a facility, a representative selection of the health facilities with active CHWs is presented in Figure 4.

Figure 3A is an example of a health facility in an active focus of transmission without CHWs. The raw PCD data for testing and reported cases are reported almost consistently throughout the time series, with two minor gaps. Data on attendees and suspected fever are missing throughout the whole time series. As a result, the numbers of estimated cases per 10,000 is low and the precision of SSe is very high, which is evident in the narrow confidence bands throughout the time series. The *P*free is high for almost the whole time period. There is a low number of confirmed cases throughout the year, with one spike in cases that is reflected in both the estimated cases and in the dip below *P*free equal to 1. This shows how the model outputs correspond to the real-world data.

Figure 3B is an example of the impact of very low reporting at a facility on the estimated number of cases, the precision around these estimates, and the estimated *P*free. There are no data reported until month 34. Despite the lack of data recorded, when data are available, there are zero cases reported. This low number of cases is reflected in the estimated case numbers; however, there is high uncertainty around these estimates. The *P*free is above 0.5 for the whole time series but fails to reach 1, meaning that *P*free is never achieved for this facility.

Of the eight facilities that had CHWs active, two facilities reached *P*free with PCD data alone, both for a period of 2 months. When CHW data were added to the model, a total of seven (88%) facilities reached *P*free, for a range of 4–22 months. The average number of months a facility sustained *P*free was 37 (SD ±21).

In Figure 4A, PCD data are available for only 3 months at the end of the time series. The CHW data are also scarce, but they supplement data for the model, providing more information than PCD data alone. With PCD data only, the estimated cases are unrealistically high, at 3,500 per 10,000 people with very high uncertainty. The addition of CHW data to the model decreases these estimates to more realistic and precise estimates of malaria cases. This precision in estimates is also reflected in *P*free estimates; where CHW data are added, the *P*free is equal to 1.

In Figure 4B, there is strong reporting from both PCD and CHWs after month 25. The PCD data report low numbers of confirmed cases, resulting in low numbers of estimated cases and high precision around these estimates. As a result, PCD data alone generate a high estimate for *P*free, which gets close to, but does not reach, the upper threshold of 1. In this case, when CHW data are included, more confirmed cases are added. Consequently, there is an increase in the number of estimated cases and an improvement in the precision of these estimates during the time that PCD and CHW data are available. The addition of CHW data also decreases the *P*free, as these data provide evidence of confirmed cases in the catchment population.

### Impact of CHW on model precision.

The average *P*free, the range in credible interval of precision of malaria case estimates, and the difference between these for health facilities with PCD plus CHW data are presented in Table 2. The average difference in the precision of model estimates for all health facilities with CHW data available was −2,061. These values ranged from −11,490 to 13. Larger differences in precision represented a larger impact of CHW data on model estimates. The addition of CHW data improved the precision in seven (88%) health facilities with CHW data. Therefore, the addition of CHW data generally improved the precision of model estimates. The overall difference in precision was negative for one health facility (Figure 4B), corresponding to an increase in variation around the estimation, or a loss of precision when CHW data were added.

**Table 2:** Average values over the survey period for PFree, the range in credible interval for malaria case estimations, the change in the range of credible interval (precision), and the quantity of change in the range of credible interval

Health Facility Designation	Average PFree		Average Credible Interval Range		Change in Precision with CHW	Fold Change in Precision with CHW
	PCD	PCD + CHW	PCD	PCD + CHW		
02	0.00	0.39	1,795.57	2.09	−1,793.48	859.12
03	0.04	1.00	526.95	0.14	−526.81	3,763.93
05	0.03	0.99	283.22	0.16	−283.06	1,770.13
06	0.03	0.98	11,489.88	0.30	−11,489.58	38,299.60
18	0.76	0.01	6.41	19.17	12.76	0.33
24	0.61	0.24	965.75	8.19	−957.56	117.92
26	0.04	1.00	450.34	0.13	−450.21	3,464.15
33	0.01	0.97	998.10	0.84	−997.26	1,188.21
Average	0.19	0.70	2,064.53	3.88	−2,060.65	532.10

CHW = community health worker; PCD = passive case detection; PFree = probability of freedom from malaria infection.

The quantity of change, or fold change, is also presented in Table 2. This was calculated as the range in credible interval for model estimates with PCD data only divided by the range in credible interval for model estimates with PCD plus CHW data. The average fold change in precision for health facilities when CHW data were added was 532.1, meaning that model estimates were on average 532 times more precise when CHW data were included. The health facilities included in Table 2 are those that had both PCD and CHW data available. Where the difference in precision is negative, the estimates of precision have improved. Conversely, where the difference in precision is positive, there has been a loss in precision of the estimates.

## DISCUSSION

We describe the successful application of the FFI framework in the Dominican Republic, including the integration of CHW data into the model. We show that on average the addition of CHW data to PCD data improves the precision of estimates more than 500-fold and demonstrates the near absence of malaria in several facility catchment populations. Additionally, we identified logical key health system parameters that can be used to improve system performance, such as the availability of testing and drugs. These parameters impact the likelihood that an individual will attend a health facility, be suspected of malaria by a health care provider, and be tested for malaria. The findings and further applications of the FFI framework will allow for more targeted efforts toward areas identified with poorer surveillance sensitivity. Together, these components can hasten progress toward the goal of elimination.

The integration of CHW data into the models allowed for the quantification of the impact of this type of ACD on model estimations. We hypothesized that CHW data added to PCD data would provide a “boost” to the estimation of *P*free and improve the precision of model estimates. Overall, *P*free was achieved more quickly and for longer periods at health facilities when CHW data were added to the models, and the precision of estimates was also improved in seven of eight health facilities. However, at the individual health facility level, we found that the inclusion of CHW data can impact *P*free and the precision of model estimates in both directions, with the scale of the impact depending on the quality of data available at the health facility. For example, in the case of one health facility, the estimated *P*free decreased and there was a loss of precision around estimated malaria cases when CHW data was added. This can be explained by the CHW data including additional confirmed malaria cases, thus decreasing the likelihood of *P*free in the catchment. Such insight could be leveraged to target additional surveillance efforts where cases are potentially being missed by PCD.

The novel methods developed during this study to integrate ACD CHW data into the FFI framework can be applied to other types of malaria data, to investigate their impacts on SSe and *P*free. For example, in settings where the results of cross-sectional surveys are available within the time frame being analyzed, survey results may provide valuable information on case numbers and malaria prevalence at a specific timepoint. Mobile health facilities and other additions to malaria surveillance systems could also act as additional sources of data that could be integrated into the FFI framework and improve precision of estimates where confirmed cases are very low, similar to the CHW data.

The results of the analysis for parameters impacting the different steps of the care-seeking cascade produced some important findings that, if implemented by control programs, could strengthen the surveillance system. Some of the factors which were the most influential for multiple steps in the cascade included the availability of testing and treatment supplies, with stockouts of supplies having a negative association with the relevant probabilities. Therefore, improvements along the supply chain would likely have a direct impact on the care-seeking behaviors of catchment populations and testing probabilities of facilities, improving the overall sensitivity of the health system. Similar findings were highlighted by Kirui et al.,^19^ who described the impact of supply chain on improvements to the delivery of malaria testing and treatment by CHWs in Kenya. Additionally, our findings support the importance of maintaining regular training around malaria testing for personnel working in the health sector, especially those working in the areas of lowest endemicity. This has been highlighted as a critical requirement for malaria elimination worldwide.^20^ Increased travel time to the health facility was associated with a decrease in the probability of care-seeking. Travel time has also been found to be a significant factor impacting care-seeking in several elimination settings, including Indonesia^14^ and Malaysia,^21^ and is often found to be a strong predictor when modeling malaria prevalence and risk factors for controlling burden.^22^^–^^27^ Where access to health facilities is limited, these results suggest that the sensitivity of malaria surveillance could be improved by CHWs performing supplementary ACD to detect any potential cases in the community.^14^ These findings validate that the model is picking up known phenomena in malaria surveillance. Other factors associated with a strong SSe included having guidelines available for reference and regular training on laboratory practices, highlighting the need to maintain high testing and training standards in health facilities as transmission continues to decline and fewer positive cases are identified.

The Dominican Republic has reported strong reductions in malaria and high-performing health areas, yet we identified some opportunities for improvement in the surveillance system, which, if implemented, could help the certification process and speed progress toward achieving elimination. Maintaining high testing levels and strong records will be critical as cases decrease and the country prepares to apply for certification. One challenging aspect of data collection was the multiple streams and systems where data are recorded and stored, with data from health facilities and CHWs following different paths. We propose establishing a unified repository for malaria record collection, along with improving alignment between surveillance bodies. Each would be a key factor in facilitating progress toward elimination. This echoes the findings of the WHO Malaria Elimination Oversight Committee (MEOC) in 2021.^3^ We are aware that development has commenced on plans to integrate the multiple streams into the national surveillance system.

There were some important limitations in this study. The FFI modeling framework relies on some essential components of the health system to make accurate estimations of the SSe and *P*free. One of these components is the catchment population, which was not regularly calculated or updated. To ensure that the data for health facilities where key variables were missing were still useful, steps were taken to infer the most likely values and to ensure that these facilities could be used in the analysis. While the FFI model results for these facilities are still important to demonstrate the impact of surveillance data on malaria health system sensitivity, these results should be interpreted with caution. Another notable limitation was the team’s understanding of the system for recording PCD. These data are usually stored as paper records at the health facility, but some are stored at the Provincial Health Directorate (DPS) and the Health Area Directorate (DAS). The data collection team was not aware of the records stored at the DPS and DAS during the period of data collection, and therefore, these records were omitted from this study, with missing values inputted for data that may have been physically available elsewhere. The malaria FFI model was developed within a spatial context, and it can account for some missing data with limited implication for the resulting estimates. However, as is seen in the results of the data available and extracted at the time of the health facility visits, too much missing data resulted in high variation and unrealistic estimations of malaria infections. For example, in many of such facilities, over 1,000 cases per 10,000 people in the catchment were estimated. If this level of transmission were truly occurring, this would be detected and reflected in the records. For the same reasons, the precision around these estimates was also highly variable between facilities. These estimations and their precision are largely an artifact of the model struggling to work with minimal data inputs. In settings where PCD data are truly patchy, utilizing alternative diagnostics with varying sensitivity and duration of relevance, such as serology, has the potential to amplify the added benefits observed from integrating ACD data into the model. Serology measures antibody responses which reflect previous exposure to malaria antigens and provides added levels of insight into ongoing and historic transmission. Population-level serological surveys offer an estimation of the number of cases over a wider time frame than a single cross-sectional survey using RDT or polymerase chain reaction diagnostics and could be used to provide further insight into residual transmission patterns.^28^ Nevertheless, this work provides an important application of the FFI model and the value added when the CCM surveillance component is incorporated to support inferences.

In conclusion, this study presents the first application of the FFI model framework where CHWs are used to supplement routine malaria surveillance. To accomplish this, we developed a new methodology for integrating CHW data into the model and quantitatively assessing its impact on SSe and *P*free. Some challenges were faced in data collection and availability of data to answer specific questions. However, overall, we demonstrate a validation of the FFI methods incorporating both PCD and CHW data. Future steps should include expansion to more facilities and provinces within the Dominican Republic, proactively identifying where data are stored for record extraction to minimize inputting missing data into the models, reapplying the FFI framework with more complete records where data availability is low, applying species-specific adjustments to the model where the data can be disaggregated by species, and determining how the results can be applied to complement existing guidelines and support the Dominican Republic’s progress toward malaria elimination.

## Supplemental Materials

10.4269/ajtmh.24-0404 Supplemental Materials
