# Supplementary material for: Incorporating Community Case Management in Risk-Based Surveillance for Malaria Elimination in the Dominican Republic
Source: Am J Trop Med Hyg. 2025 Jan 21;112(4):775–83. doi: 10.4269/ajtmh.24-0404 (PMC11965756; doi:10.4269/ajtmh.24-0404)
Supplement: Supplemental Materials [file tpmd240404.SD1.pdf]

Supplemental Information

Supplementary Information Appendix 1:

The full description of the Freedom From Infection (FFI) framework, which underpins the statistical model used in this study, is available in detail in Nelli et al. (2023). This supplementary material provides a concise overview of the key components and adaptations of the FFI model as applied in this study, particularly in integrating Community Health Worker (CHW) data to enhance malaria surveillance in the Dominican Republic. Please refer to Nelli et al. (2023) for full details.

The FFI model framework is designed to assess the Surveillance System Sensitivity (SSe) and the Probability of Freedom from Infection (PFree). These measures are crucial for evaluating the effectiveness of malaria surveillance systems, especially in low-transmission settings.

The FFI model integrates two key processes: a **state process**, which estimates the true number of malaria infections, and an **observation process**, which models the likelihood of these infections being detected by the health system.

<u>State Process: Modelling Malaria Infections</u>

We consider the catchment regions of *J* health facilities and a passive case detection (PCD) longitudinal series of *I* surveillance months. We assume that the total number ($M_{i,j}$) of real malaria infections for the ith month and the catchment area of the jth health centre, is given by the sum of endemic infections (i.e., those that originated in the catchment population, $E_{i,j}$) and imported infections (i.e., those that originated in any other catchment area within our outside of the study site $O_{i,j}$).

$$M_{i,j} = E_{i,j} + O_{i,j}$$

We modelled endemic infections as a Poisson process,

$$E_{i,j} \sim Poisson(\lambda_{E_{i,j}} N_{i,j})$$

whose rate is the product of $N_{i,j}$, the catchment population and $\lambda_{E_{i,j}}$, the force of infection related to the proportion of individuals in the population who are expected to have malaria at any given time. Epidemiological dynamics of endemicity are approximated statistically by a 2nd order autoregressive model, together with an error term ($\varepsilon_{i,j}$) generating extra-Poisson dispersion in the process.

$$log\left(\lambda_{E_{i,j}}\right) = \alpha_{0\,j} + \alpha_1 M_{(i-1),j} + \alpha_2 M_{(i-2),j} + \varepsilon_{i,j}$$

Where $\exp(\alpha_0)$ determines the expectation of endemic infections at any time when no infections have been observed for two-time lags. α_1 and α_2 regulate temporal dependencies (please see Nelli et al. for further details).

We modelled incoming infections as a Poisson

$$O_{i,j} \sim Poisson(\lambda_{O_{i,j}})$$

with rate determined by neighboring infection rates, weighted by geographical proximity process (please see Nelli et al. for further details)

Observation Process: Modelling Malaria Detection

For the ith month and j^{th} health centre, we collected data on the number of patients attending the facility ($A_{i,j}$), the number of people reporting fever ($F_{i,j}$), the number of people tested for malaria ($T_{i,j}$), and the number confirmed cases ($C_{i,j}$). We structured the observation process as a causal chain of events leading from $M_{i,j}$, the unobserved cases present in each sub-population to $C_{i,j}$, the malaria cases ultimately being detected by the health system.

We modelled the number of patients attending the health facility as resulting from a Poisson process:

$$A_{i,j} \sim Poisson\left(\lambda_{\alpha_{i,j}}\right)$$

with the rate $\lambda_{\alpha_{i,j}}$ is given by

$$\lambda_{\alpha_{i,j}} = \left(r_j N_{i,j} + P_{CLINICAL} M_{i,j}\right) P_{SEEK_j}$$

Where $N_{i,j}$ is the catchment population, r_j is the background monthly proportion of ill people (i.e., people who attend the health facility for all reasons other than malaria) in each catchment population, $P_{CLINICAL}$ is the proportion of symptomatic malaria infections, and P_{SEEK_j} is the probability of care-seeking at each health facility.

We modelled the number of people with fever symptoms as a Poisson process:

$$F_{i,j} \sim Poisson\left(\lambda_{FEV_{i,j}}\right)$$

with the rate $\lambda_{FEV_{i,j}}$ given by

$$\lambda_{FEV_{i,j}} = s r_j N_{i,j} + P_{CLINICAL} M_{i,j}$$

Where s is the proportion of people with any pyretic disease other than malaria.

When modelling the number of patients tested for malaria, we assume that this can result from the rate $\lambda_{FEV_{i,j}}$, weighted by the probability of being tested P_{TEST_j}, according to:

$$T_{i,j} \sim Poisson\left(P_{TEST_j} \lambda_{FEV_{i,j}}\right)$$

Finally, we modelled the number of patients confirmed with malaria as

$$C_{i,j} \sim Poisson\left(\lambda_{CONF_{i,j}}\right)$$

with the rate $\lambda_{CONF_{i,j}}$ defined as

$$\lambda_{CONF_{i,j}} = P_{CLINICAL} P_{TEST_j} M_{i,j}$$

Freedom From Infection

We modelled the latent state and observation processes simultaneously in an integrated way. We used the joint posterior distribution of all imputed values of $M_{i,j}$ (across time and regions) to calculate the probability of freedom from infection at a given time point and for a given threshold. We achieved this by calculating what percent of posterior density at any given time and placer falls below the threshold. For example, here we defined PFree as the probability of having achieved elimination at a threshold of less than 1 infection every 10,000 people (ranging between 0 and 1), by measuring how much of the posterior distribution of $M_{i,j}$ is under 1 infection every 10,000.

Parameter Estimation and Covariate Modeling

In this study, the parameters were estimated using a mix of expert-based priors, literature-informed priors, and empirical data. The probability of care-seeking P_{SEEK} and the probability of being tested for malaria P_{TEST}) were modelled as a function of covariates related to health facility characteristics, such as the availability of antimalarial drugs, stock-outs, and accessibility. These covariates were included in the model to capture the variability in surveillance sensitivity across different health facilities. For a comprehensive description of the modelling approach, including the full details on parameter estimation and the specific covariates used, please refer to Nelli et al. (https://doi.org/10.21203/rs.3.rs-2813944/v2).

Supplementary Information Appendix 2:

Extensions to the FFI model to integrate CHW data:

The CHW data were integrated into the FFI model to enhance the estimation of critical parameters, such as the underlying malaria transmission $M_{i,j}$, the rate of fever cases $\lambda_{\mathrm{FEV}\ \mathrm{i,j}}$, and the rate of attendance at health facilities $\lambda_{\alpha_{\mathrm{i,j}}}$. Specifically, the CHW data contributed to these estimations simultaneously with the passive case detection (PCD) data, providing a more robust and comprehensive assessment of malaria transmission and surveillance sensitivity.

The number of patients attending a CHW (A_{CHW}) was modelled as

1. $\mathrm{A}_{\mathrm{CHW}\ \mathrm{i,j}} \sim \mathrm{Pois}(\lambda_{\alpha_{\mathrm{i,j}}})$

We modelled the number of patients tested for malaria (T_{CHW}), *as:*

2. $\mathrm{T}_{\mathrm{CHW}\ \mathrm{i,j}} \sim \mathrm{Pois}(\mathrm{P}_{\mathrm{TEST}_{\mathrm{CHW}\ \mathrm{j}}}\, \lambda_{\mathrm{FEV}\ \mathrm{i,j}})$

with $\mathrm{logit}(\mathrm{T}_{\mathrm{TESTCHW}_{\mathrm{j}}})$ *being a simple function of a different intercept for each CHW. Finally, we modelled the number of patients confirmed with malaria as*

3. $\mathrm{C}_{\mathrm{CHW}\ \mathrm{i,j}} \sim \mathrm{Pois}(\lambda_{\mathrm{CONF}_{\mathrm{CHW}_{\mathrm{i,j}}}})$

With the rate $\lambda_{\mathrm{CONF}\ \mathrm{CHW}_{\mathrm{i,j}}}$ *defined as*

4. $\lambda_{\mathrm{CONF}\ \mathrm{i,j}} = \mathrm{P}_{\mathrm{CLINICAL}} \mathrm{P}_{\mathrm{TEST}\ \mathrm{CHW}\ \mathrm{j}} \mathrm{M}_{\mathrm{i,j}}$

Note that all these steps of the CHW-related observation process, share the same estimation of the underlying malaria transmission ($\mathrm{M}_{\mathrm{i,j}}$). *In this way, in the health facilities where CHW were active, in addition to standard surveillance, we could obtain two different estimates of* $\mathrm{M}_{\mathrm{i,j}}$ *and* $\mathrm{PFree}_{\mathrm{i,j}}$ *(one with health facility data alone, and one with health facility plus CHW data).*

Supplementary Information Table 1: Health system interview questionnaire

**Care-seeking**	
	Approximately how many people reside in the catchment area of this facility?
	What is the estimated average monthly number of outpatients attending your health facility?
	Approximately, what percentage (%) of patients reporting at your facility are from outside your catchment area?
	What municipality are most patients who live outside the catchment area resident in?
	Approximately, what percentage (%) of patients reporting at your facility have travelled outside of the country in the past 4 weeks?
	Approximately, what percentage (%) of all health facility attendees reported fever in the last month?
	Among those with a fever, approximately what percentage (%) were tested for malaria? (Microscopy or RDT)
	Approximately, what percentage (%) of health facility attendees that are 15 years of age or younger reported fever in the last month?
	Approximately, what percentage (%) of health facility attendees that are over 15 years of age reported fever in the last month?
**Malaria testing**	
	Is malaria diagnosed by microscopy at this facility?
	Have you experienced stock-outs of microscopy supplies in the past 12 months?
	If yes, how many months were supplies out of stock?
	Is malaria diagnosed using RDT at this facility?
	If yes, have you experienced stock-outs of RDT supplies over the past 12 months?
	If yes, how many months were RDT supplies out of stock?
**Antimalarial drugs**	
	Does your facility provide antimalarial treatment?
	If yes, has your facility experienced stock-outs of antimalarials in the last 12 months?
	If you experienced a stockout, how many months were supplies out of stock?

	Is a copy of the national malaria treatment guidelines or standard operating procedures on malaria case management available in your facility?
**Malaria Testing Capacity**	
	Do you have staff available to conduct malaria testing at the facility?
	Have your staff received training on malaria diagnosis in the last 2 years?
	Have your staff had a training or a workshop on malaria case management in the last 2 years?
	Have you had a supervision visit in the last year?
	If microscopy testing is provided at this facility, has microscopy proficiency panel testing been conducted in the last year?
	What were the results of the last microscopy proficiency panel testing?
	Does your facility conduct routine monthly cross-checking of microscopy slides with the reference laboratory?
**Malaria reporting**	Do you normally record the number of patients suspected for malaria per month?
	Do you normally record the number of patients tested for malaria per month?
	Do you normally record the number of patients confirmed for malaria per month?
	How many months in the last 12 months did you report the monthly malaria data to the national database?
**Malaria training**	
	Did your facility receive training on data entry into the national database in the last year?
	What is the case definition for an imported case for this facility?
	What is your case definition/ criteria to test a patient for malaria?
	Describe the investigation process you use to confirm if a case is imported or indigenous?
	If you find a malaria infection caused by *P. vivax* or *P. ovale*, please describe how you identify whether this is a new infection or a relapse?
	Is there someone at this facility who is trained to conduct a malaria foci investigation?

Supplementary Information Table 2: Result of Bayesian models for probability of care seeking, probability of being suspected and tested for malaria, as a function of covariates obtained through questionnaires at health facilities. β: mean of posterior distribution, sd: standard deviation, LCI: lower credible interval, UCI: upper credible interval. Variables in bold have posterior distributions which do not span 0.

Probability	Variable	Beta	LCI	UCI
ATTENDING	**RDT provided**	**0.523**	**0.346**	**0.743**
	RDT stockout	-0.035	-1.030	0.794
	Antimalarials provided	-0.472	-1.525	0.494
	**Antimalarials stockout**	**-0.626**	**-1.229**	**-0.108**
	Case training	-0.466	-3.016	1.555
	Competency assessment	22.752	-10.739	66.449

	Microscopy cross checking	0.048	-0.657	0.546
	**Microscopy proficiency training**	**6.510**	**1.513**	**15.914**
	**Record confirmed cases**	**0.241**	**0.054**	**0.435**
	**Record suspected cases**	**0.483**	**0.085**	**0.911**
	Record tested	0.199	-0.031	0.393
	Supervision visit	-0.683	-0.837	0.542
	**Travel time to facility**	**-0.069**	**-0.135**	**-0.025**
	Staff available to test	0.280	-0.301	0.752
	Staffed trained to test	-0.261	-0.860	0.111
	Treatment guidelines available	0.321	-0.103	0.664
**SUSPECTED**	RDT provided	-1.334	-3.457	0.020
	RDT stockout	0.416	-1.657	3.846
	Antimalarials provided	0.105	-1.524	1.103
	Antimalarials stockout	-0.424	-2.002	1.052
	Case training	-1.769	-2.905	0.007
	Competency assessment	0.921	-0.467	2.257
	Microscopy provided	-1.243	-2.509	0.841
	Microscopy stockout	-2.226	-2.983	-1.559
	**Microscopy cross-checking**	**1.019**	**0.643**	**1.581**
	Microscopy proficiency training	-1.160	-1.753	-0.640
	Record confirmed cases	-0.017	-0.249	0.273
	Record suspected cases	-0.335	-0.845	0.315
	Record tested	0.043	-0.361	0.314
	Supervision visit	0.711	-0.316	1.387
	Staff available to test	-0.161	-0.517	0.163
	Staff trained to test	-0.542	-1.230	0.209
	Treatment guidelines available	-0.183	-0.385	0.091
**TESTED**	**RDT provided**	**3.668**	**1.445**	**5.375**
	**RDT stockout**	**-1.547**	**-2.157**	**-0.984**
	Case training	-0.971	-3.118	2.103
	Competency assessment	11.920	-0.577	31.363
	**Microscopy provided**	**2.112**	**0.840**	**3.650**
	Microscopy stockout	-0.190	-2.535	3.206
	Microscopy cross-checking	0.310	-1.590	2.943
	Microscopy proficiency training	0.116	-2.040	1.747
	Record confirmed cases	-0.329	-1.138	0.657
	Record suspected cases	0.274	-0.457	1.040
	Supervision visit	0.099	-3.700	1.898
	Staff available to test	-0.654	-2.135	0.821
	Staff trained to test	0.211	-2.108	3.986
	**Treatment guidelines available**	**2.585**	**1.133**	**4.185**

References

L, N. et al. (2023) “Freedom From Infection (FFI): A paradigm shift towards evidence-based decision-making for malaria elimination.” Available at: https://doi.org/10.21203/RS.3.RS-2813944/V1.
